# The Role of Optical Coherence Tomography in Differential Diagnosis of Multiple Sclerosis and Autoimmune Connective Tissue Diseases with CNS Involvement

**DOI:** 10.3390/jcm9051565

**Published:** 2020-05-21

**Authors:** Paula Wildner, Ewa Zydorczak, Magdalena Oset, Małgorzata Siger, Michał Wilczyński, Mariusz Stasiołek, Mariola Matysiak

**Affiliations:** 1 Department of Neurology, Medical University of Lodz, 90-414 Lodz, Poland; paula.wildner@gmail.com (P.W.); magdalena.oset@gmail.com (M.O.); malgorzata.siger@umed.lodz.pl (M.S.); mariola.swiderek-matysiak@umed.lodz.pl (M.M.); 2 Department of Ophthalmology, Medical University of Lodz, 90-414 Lodz, Poland; ewa.zydorczak@gmail.com (E.Z.); michal.wilczynski@umed.lodz.pl (M.W.)

**Keywords:** optical coherence tomography, multiple sclerosis, autoimmune connective tissue diseases, rheumatic disorders, differential diagnosis

## Abstract

The purpose of this study was to examine whether application of optical coherence tomography (OCT) measurements can provide a useful biomarker for distinguishing central nervous system (CNS) involvement in autoimmune connective tissue diseases (CTD) from multiple sclerosis (MS). An observational study included non-optic neuritis eyes of 121 individuals: 59 patients with MS, 30 patients with CNS involvement in CTD, and 32 healthy controls. OCT examination was performed in all subjects to measure retinal nerve fiber layer (RNFL) thickness, ganglion cell complex (GCC) thickness, ganglion cell layer-inner plexiform layer (GCIPL) thickness, and volume of the macula. There was a significant group effect with regard to superior optic disc RNFL, macular RNFL, GCC, and GCIPL thickness, and macular volume. Post-hoc analysis revealed that MS patients have significantly smaller macular volume and thinner superior optic disc RNFL, macular RNFL, GCC, and GCIPL compared to healthy controls. CTD patients have significantly smaller superior optic disc RNFL, GCIPL, and GCC thickness compared to healthy controls. However, no significant group differences were observed between the patient groups (MS vs. CTD) on any outcome. Although a prominent retinal thinning may be a useful biomarker in MS patients, in a general population of individuals with a confirmed CNS involvement the use of OCT is not specific enough to discriminate between MS and autoimmune CTD.

## 1. Introduction

Multiple sclerosis (MS) is presumed to be the most prevalent acquired demyelinating disorder of the central nervous system (CNS) [1]. Diagnosing MS might be complex and demanding, due to its diverse clinical features and the lack of fully specific findings. Among numerous disorders, autoimmune connective tissue diseases (CTD) with CNS involvement constitute a spectrum which apparently should be taken into consideration in the process of differential diagnosis. Despite a broad research, currently, no fully specific diagnostic test is accessible to distinguish between MS and other CNS inflammatory disorders. Although magnetic resonance imaging (MRI) of brain and spinal cord remains crucial for this process, in some cases it may be a source of confusion, as radiological findings may overlap, e.g., in CTD and MS. A combination of clinical examination, MRI and laboratory tests remains currently mandatory to ascertain a right diagnosis. Recently, optical coherence tomography (OCT) has been proven as a valuable noninvasive tool to assess neurodegenerative features in MS [2]. The cascade of MS pathology comprises demyelination, oligodendrocyte loss, axonal damage, neuronal loss, astrogliosis, and progressive failure of remyelination [3,4]. All these processes coexist at every stage of the disease, and the progressive phase of MS becomes clinically visible when the axonal damage threshold is surpassed [5]. Available data indicate that axonal injury is playing a pivotal role in the accumulation and persistence of neurological deficits [6,7]. It has been shown that peripapillary retinal nerve fiber layer (RNFL) thinning observed with the use of OCT may serve as a marker of neurodegeneration in MS patients [8]. It has been suggested that especially the temporal thickness of RNFL can be related to physical disability [9]. Until now, the application of OCT in the assessment of other inflammatory CNS diseases has not been sufficiently established. Recently, RNFL thinning observed in patients with neuromyelitis optica spectrum disorders (NMOSD) was associated with spreading of inflammatory activity. The same longitudinal study revealed macular ganglion cell-inner plexiform layer (GCIPL) reduction over time in all NMOSD patients, as well as in all progressive MS (PMS) and active relapsing-remitting MS (RRMS) patients, but not in stable RRMS patients [10]. Another study showed GCIPL thinning in AQP4-IgG-seropositive NMOSD patients over time [11], in contrast to a group of MOG-IgG-seropositive NMOSD patients in which progression of GCIPL reduction was not observed [12]. Importantly, a study comprising patients with systemic lupus erythematosus (SLE), demonstrated significant differences in RNFL and macular thickness between SLE patients and healthy controls and a positive correlation of cognitive performance and temporal RNFL thickness. However, patients with neuropsychiatric SLE (NPSLE) did not differ significantly from SLE patients [13].

Taking into consideration recent findings, incorporation of OCT in the differential diagnosis of CNS inflammatory diseases seems to represent a promising approach. However, to our knowledge as yet no analysis evaluating an applicability of OCT in the differential diagnosis of MS and rheumatic diseases with CNS involvement has been performed. The purpose of this study was to examine whether OCT parameters measurements can provide a useful biomarker for distinguishing CNS involvement in autoimmune CTD from MS.

## 2. Experimental Section

### 2.1. Study Design

Patients with a confirmed diagnosis of MS or an autoimmune CTD with CNS involvement were recruited consecutively, promptly after or during the diagnostic process, between July 2018 and October 2019 in the Neurology Department of the Medical University of Lodz, Poland. In the MS group, all subjects fulfilled the 2017 McDonald diagnostic criteria for MS and had a relapsing-remitting disease phenotype Only patients with a definite diagnosis were included in this study. Both the MS and the CTD groups comprised exclusively patients, in which MRI examination had been performed formerly in the diagnostic process and revealed demyelinating-type lesions raising suspicion of MS. Cerebrospinal fluid (CSF) examination was performed in majority of patients to ascertain the diagnosis. We excluded from the MS study group patients with questionable CSF findings such as protein concentration above 100 mg/dL, pleocytosis over 50 cells/mm^3^, presence of atypical cells. CSF examination in some cases was performed also to exclude viral or paraneoplastic process. Patients younger than 18 years and older than 50 years were excluded from the study. Exclusion criteria encompassed also subjects with any other conditions which may affect RNFL thickness (such as glaucoma, pathological retinal findings on ophthalmologic examination, a refractive error of +/− 6D), as well as subjects with opacities of the ocular optical media. Eyes with a history of optic neuritis (ON) were excluded based on anamnesis and ophthalmological examination. The criteria used to exclude eyes with a history of ON were the following: History of pain during eye movement, acute loss of visual acuity and improvement in the further course of the disease, impaired color perception. Ophthalmological examination including best-corrected visual acuity, intraocular pressure, color perception, pupillary light reflex, slit lamp examination of anterior and posterior segment of both eyes was performed in all subjects and eyes with significant abnormal findings were excluded from the study group. For patients in which a visual evoked potential (VEP) test had been performed in the diagnostic process, its results were also considered. Eyes with P100 amplitude reduction and P100 latency delay were excluded as suspected of ON. OCT examination data of healthy controls came from the database of the Ophthalmology Department. Healthy controls had negative history of ophthalmologic and neurologic diseases, were age- and sex-matched. This was an observational study. Ethical approval of the study was given by the Local Ethics Committee of Medical University of Lodz (approval number RNN/231/18/KE, 12 June 2018).

### 2.2. Data Collection

Spectral domain optical coherence tomography (SD-OCT) examination (REVO NX SOCT, software version 9.5.0; Optopol Technology, Zawiercie, Poland) was performed in all subjects to measure RNFL thickness, ganglion cell complex (GCC) thickness, ganglion cell layer-inner plexiform layer (GCIPL) thickness, and volume of the macula. 

RNFL scans were performed using a preset protocol launched by the OCT user interface. The examined eye was fixated on an internal light and a high-speed circle scan of 3.40 mm diameter and circle thickness of 0.55 mm, centered on the optic nerve head was performed.

The optic disc RNFL thickness was measured as an average thickness across four segments: Superior (120 degrees), temporal (50 degrees), inferior (120 degrees), and nasal (70 degrees). The average RNFL thickness was calculated also for the papillomacular bundle. The computer software maps all layers, including the two borders of the RNFL, and automatically calculates the thickness of the layers. Macular volume was automatically measured by the software provided by the manufacturer. The computer software takes 128 horizontal B-scans covering a field of 7 × 7 mm. Any scans with a quality index (QI) of less than 7 were excluded (maximum value accounted to 10). 

Information gathered from patients included age, time from the first neurological symptoms, concomitant diseases. 

### 2.3. Statistical Analysis 

OCT measurements were performed for each non-optic neuritis (non-ON) eye of each subject, without any differentiation between the right and the left eye. Analyses were performed with Statistica 13, StatSoft (Cracow, Poland). Non-parametric tests have been implemented as both the normality of data distribution and the homogeneity of variance assumptions were violated, as revealed by Shapiro–Wilk test and Levene’s test, respectively. Kruskal–Wallis test was used to assess the differences in OCT measurements among study groups (MS patients, patients with CTD with CNS involvement, healthy controls). The Bonferroni correction for multiple testing was used when appropriate. U Mann–Whitney test was performed to compare MS patients and patients with CTD with CNS involvement. 

## 3. Results 

### 3.1. Participants Characteristics and Demographics

The study included a total of 121 individuals divided into three groups: patients with MS (*n* = 59), patients with CNS involvement in CTD (*n* = 30), and healthy controls (*n* = 32). The majority of patients with CTD were diagnosed with systemic lupus erythematosus (SLE) with or without secondary antiphospholipid syndrome (APS) (8 patients). Other diagnoses in this group were: Sjogren’s syndrome (3 patients), neurosarcoidosis (2 patients), rheumatoid arthritis (3 patients), hypereosinophilic syndrome (2 patients), psoriasis (1 patient), juvenile idiopathic arthritis (1 patient), CNS vasculitis (1 patient), and undifferentiated connective tissue disease (9 patients). MS group included: 47 patients without any concomitant disease and 12 patients with concomitant diseases: Hypothyroidism (4 patients), diabetes mellitus (3 patients), asthma (3 patients), endometriosis (1 patient), and autoimmune hepatitis (1 patient). All the subjects in MS and CTD groups had white matter lesions in brain MRI. In the MS group, radiological findings were consistent with the 2017 McDonald diagnostic criteria for MS. In the CTD group, demyelinating white matter lesions were present in all the patients. Additional brain lesions suggestive of ischemic infarcts were revealed in MRI of one patient in this group. As all patients were recruited consecutively promptly after or during the diagnostic process, patients were not exposed to immunomodulatory treatment. Table 1 lists age, gender, time from the occurrence of first neurological symptoms in each group.

### 3.2. Retinal Nerve Fiber Layer Thickness and Macular Volume

**Figure 1:**
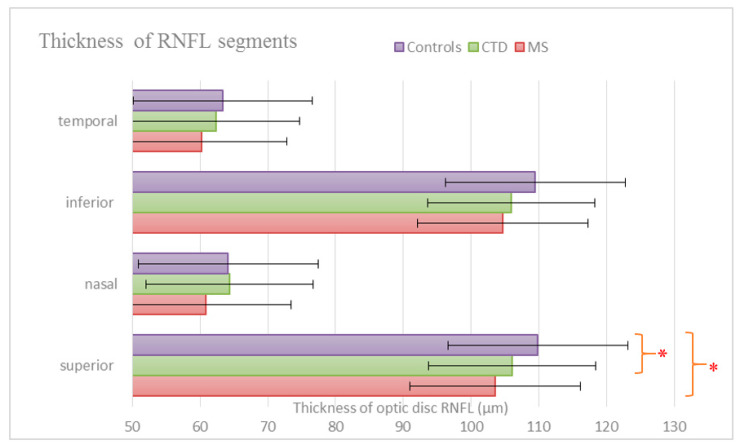
Mean values of optical coherence tomography (OCT) measurements of optic disc retinal nerve fiber layer (RNFL) thickness according to segments. There was a significant group effect with regard to superior optic disc RNFL thickness (*p* = 0.0202). Post-hoc analysis revealed that MS patients have significantly thinner superior optic disc RNFL compared to healthy controls (two-sided *p*-values with a Bonferroni adjustment: 0.0176). Connective tissue diseases (CTD) patients have significantly smaller superior optic disc RNFL compared to healthy controls (*p* = 0.0456). No significant group differences were observed between the patient groups (MS vs. CTD) in abovementioned parameters. Statistically significant differences in the post-hoc analysis are indicated (* *p* < 0.05).

**Figure 2:**
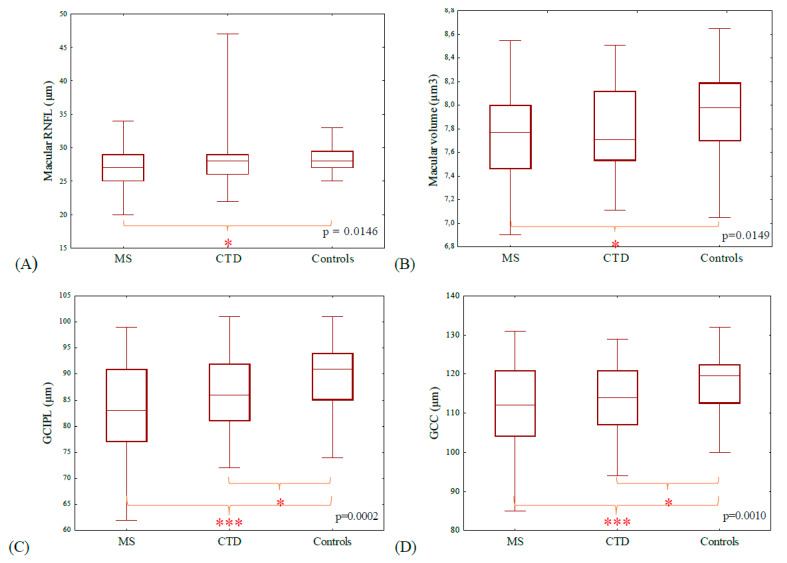
There was a significant group effect with regard to macular RNFL thickness (**A**), macular volume (**B**), ganglion cell layer-inner plexiform layer (GCIPL) (**C**), and ganglion cell complex (GCC) (**D**) thickness (Kruskal–Wallis analysis *p*-values shown in the figure, respectively: 0.0146, 0.0149, 0.0002, and 0.0010). Post-hoc analysis revealed that MS patients have significantly lower abovementioned parameters compared to healthy controls (respectively two-sided *p*-value with a Bonferroni adjustment: 0.0163, 0.0129, 0.0001, and 0.0006). CTD patients have significantly smaller GCIPL, and GCC thickness compared to healthy controls (respectively *p* = 0.0222 and *p* = 0.0280). No significant group differences were observed between the patient groups (MS vs. CTD) in abovementioned parameters. Statistically significant differences in the post-hoc analysis are indicated (* *p* < 0.05, *** *p* < 0.001).

Our study showed significant differences in RNFL parameters of non-ON eyes in the three study groups (Table S1). There was a significant group effect regarding superior optic disc RNFL (*p* = 0.0202) (Figure 1), and macular RNFL (*p* = 0.0146) (Figure 2). Post-hoc analysis revealed that MS patients have significantly thinner superior optic disc RNFL and macular RNFL compared to healthy controls (two-sided *p*-values with a Bonferroni adjustment: 0.0176 and 0.0163, respectively). CTD patients have significantly smaller superior optic disc RNFL compared to healthy controls (*p* = 0.0456). However, no significant group differences in abovementioned parameters were observed between the patient groups (MS vs. CTD). No significant difference was observed in average optic disc RNFL thickness, and temporal, inferior and nasal segments of optic disc RNFL among the three groups.

The study revealed a significant group effect in macular volume (*p* = 0.0149). Post-hoc analysis revealed that MS patients have significantly smaller macular volume in comparison with healthy controls (two-sided *p*-value with a Bonferroni adjustment: 0.0129). No significant group difference was observed between the patient groups (MS vs. CTD). Macular volume was not statistically different between the healthy control and CTD patients (*p* = 0.0555) (Figure 2). 

### 3.3. Ganglion Cell Complex (GCC) and Ganglion Cell Layer-Inner Plexiform Layer (GCIPL)

Analysis of non-ON eyes demonstrated a significant difference in GCC and GCIPL thickness among all study groups. There was a significant group effect with regard to GCC and GCIPL thickness (respectively *p* = 0.0010 and *p* = 0.0002) (Figure 2). Post-hoc analysis revealed that MS patients have significantly thinner GCC, and GCIPL compared to healthy controls (respectively two-sided *p*-value with a Bonferroni adjustment: 0.0006 and 0.0001). CTD patients have significantly smaller GCC, and GCIPL thickness compared to healthy controls (respectively *p* = 0.0280 and *p* = 0.0222). Again, no significant group differences were observed between the patient groups (MS vs. CTD) in abovementioned parameters. 

### 3.4. OCT Parameters in the Subgroups of CTD Patients

Taking into consideration the heterogeneity of the CTD group and its possible influence on the obtained results, two subgroups of patients with conventional CTDs were selected for more detailed additional analysis: SLE group (*n* = 8) and undifferentiated connective tissue disease (UCTD) group (*n* = 9). Statistical analysis performed in the four study groups (MS, SLE, UCTD, and healthy controls) has shown results concordant with the findings obtained previously (Figure S1). There was a significant group effect regarding superior optic disc RNFL, GCC, GCIPL, macular RNFL thickness, and macular volume (Kruskal–Wallis analysis *p*-values, respectively: 0.0143, 0.0016, 0.0003, 0.0129, and 0.0097). Post-hoc analysis revealed that MS patients have significantly lower values of abovementioned parameters compared to healthy controls (respectively two-sided *p*-value with a Bonferroni adjustment: 0.0228, 0.0007, 0.0001, 0.0271, and 0.0193). No significant group differences were observed between the patient groups (MS vs. SLE, MS vs. UCTD), nor between each CTD group (SLE, UCTD) and healthy controls.

## 4. Discussion 

Differential diagnosis of MS and other diseases with CNS white matter involvement, including CTDs, constitutes often a challenge in clinical practice. Thus, establishing a specific biomarker for distinguishing CNS involvement in rheumatic diseases from MS would be of primary importance. To our knowledge, this is the first study examining the applicability of SD-OCT in differential diagnosis of MS and CNS involvement in autoimmune CTD.

Our results, imply that OCT findings such as significant thinning of optic disc RNFL, GCC, GCIPL, macular RNFL, and a decrease in macular volume may be regarded as potentially useful biomarkers of MS in general population. However, there were no significant differences in any of the analyzed OCT parameters between patients with MS and autoimmune CTD with CNS involvement. This implies that among individuals with a confirmed CNS involvement, the use of OCT is not specific enough to discriminate between MS and other CNS demyelinating disorders. 

OCT has long been incorporated in the evaluation of MS patients. Our results are consistent with previous studies showing that MS patients had a significant thinning of the inner retinal layers (IRL) in comparison with healthy controls [14]. IRL is a combination of the retinal nerve fiber layer and the ganglion cell and the inner plexiform layer [15]. Remarkable differences between the eyes of people with MS and control eyes were found in the peripapillary RNFL and macular GCIPL, as described in meta-analysis of 5776 MS post ON and non-ON eyes [16]. Beginning with the earliest studies of OCT in MS, it has been observed that in patients with a history of ON, thinner peripapillary RNFL was noted both in eyes with and without prior ON, as compared with healthy controls [17]. The extent of IRL thinning in MS is greater in eyes affected by ON than in eyes without ON [14]. Interestingly, in the recent publication RNFL changes were also suggested as a predictive marker of persistent visual disability in MS patients with ON [18]. However, both patients with and without ON present a slowly progressing loss of optic nerve axons and retinal ganglion cells [19,20,21]. Importantly, peripapillary RNFL thickness has been proposed as a structural marker of neurodegeneration in MS [8]. First OCT studies focused on global RNFL thickness as a maker of axonal integrity in the retina, but current segmentation techniques have enabled detailed quantification of specific retinal segments [22]. The distribution of RNFL loss shows a predilection for the temporal quadrant [23]. In our analysis of non-ON MS patients eyes the significant optic disc RNFL thinning was observed specifically in the superior quadrant, which is consistent with earlier published report [24].

Thinning of neuroretinal layers is related to brain atrophy [2], disease activity and progression of disability in MS patients [25]. However, it should be emphasized that RNFL thinning is not pathognomonic for the optic nerve atrophy secondary to MS. All types of optic neuropathy—either ischemic, toxic, post traumatic, or inflammatory unrelated to MS—are connected with the peripapillary RNFL thinning [26]. Some of the studies suggest that the combined GCIPL OCT parameter has the strongest potential for identifying pathology related to MS [16]. GCIPL serves well as a stable parameter to evaluate retinal neuro-axonal damage [27,28]. As with peripapillary RNFL, there is evidence of subclinical loss of GCIPL thickness even without ON [19], and this parameter can be used to detect retinal damage prior to peripapillary RNFL thinning [29]. Our study has revealed significant GCC and GCIPL thinning in MS patients, which again is consistent with the prior research. Most importantly, we did not find significant difference in GCIPL thinning between MS and CTD patients. Contrary to MS, little data is currently available concerning retinal evaluation with OCT in CTD with CNS involvement. Our study demonstrated significant differences in superior optic disc RNFL, GCC, and GCIPL thickness between patients with an CTD with CNS involvement and healthy controls. The majority of the published studies evaluated patients diagnosed with NPSLE. A trend to lower absolute values of RNFL thickness in the SLE group was reported in the study by Liu and colleagues [13]. This study, comprising NPSLE and non-NPSLE patients, did not demonstrate significant differences in RNFL thickness or macular thickness between these groups. However, SLE patients had significantly thinner RNFL than controls. The authors suggested that RNFL and macular thinning in NPSLE may be due to vasculitis leading to microinfarcts, in mechanism associated with deposits of IgG immune complexes in the walls of retinal vessels, responsible for RNFL infarcts and ganglion cell atrophy [13]. Shulman and colleagues aimed to assess RNFL thickness as a biomarker of white matter damage in SLE patients. No statistically significant difference was observed neither between SLE patients and healthy controls, nor between patients with and without neuropsychiatric involvement [30]. So far, the available data concerning OCT findings in other rheumatic diseases with CNS involvement are scarce. CNS involvement in neurosarcoidosis (NS) is often subclinical. It has been demonstrated that 33% of NS patients without any ophthalmological symptoms had retinal abnormalities in OCT [31]. Significantly lower peripapillary RNFL and macular thickness were also reported in neuro Behcet disease patients when compared to healthy subjects [32]. Subclinical involvement of visual pathways has also been reported in MS with the use of various tests with different sensitivity [33]. VEP has been shown to be more sensitive than OCT in detection of clinical and subclinical ON in a group of patients with the history of ON [34]. The superiority of VEP over OCT in detection of subclinical visual impairment has also been demonstrated in MS patients without visual symptoms and with no history of ON [35]. In the context of our study, in contrast to the MS group, not all of the CTD patients underwent VEP analysis. However, the goal of our study was to evaluate the utility of OCT as a putative biomarker in MS with respect to the role of this technique in the diagnostic process. The main limitation of the study was a high heterogeneity of the CTD group, as it included patients with disorders of diverse pathogenesis and clinical course. Further research, with more homogeneous and bigger populations of patients, is warranted in order to prove our observations in the context of CNS involvement in particular CTDs. Nevertheless, our findings are based on a relatively large group of patients, examined with the unified diagnostic protocol in the real world settings, which underlines possible clinical applicability of the results. Of particular importance is the observation that OCT parameters such as RNFL and GCIPL did not differentiate MS and CTD with CNS involvement. We suggest that OCT may still be considered as a potential biomarker utilized in characterization of the disease subtype and determination of the extent of therapeutic response in MS patients. However, available OCT measures, especially when interpreted in isolation, are not specific enough for the diagnostic process of MS.

## Figures and Tables

**Table 1:** Age, gender, and time from the occurrence of first neurological symptoms in each group.

	MS Group	Connective Tissue Disease Group	Healthy Controls
Number of patients	59	30	32
Number of eyes	101	58	64
Age, years (SD)	34.88 (9.27)	39.63 (7.87)	40.41 (9.98)
Female/Male	44/15	23/7	29/3
Duration of neurological symptoms, months (SD)	20.94 (26.50)	55.00 (91.87)	-

MS—multiple sclerosis; SD—standard deviation.

## Supplementary Materials

The following are available online at https://www.mdpi.com/2077-0383/9/5/1565/s1, Figure S1: OCT parameters in the subgroups of CTD Patients, Table S1: Mean values of OCT measurements.

## Abbreviations

APS: antiphospholipid syndrome; AQP4-IgG, aquaporin-4 immunoglobulin G; CNS, central nervous system; CTD, connective tissue diseases; EAE, experimental autoimmune encephalomyelitis; GCC, ganglion cell complex; GCIPL, ganglion cell-inner plexiform layer; IgG, immunoglobulin G; IPL, inner plexiform layer; IRL, inner retinal layers; MOG-IgG, myelin oligodendrocyte glycoprotein immunoglobulin G; MRI, magnetic resonance imaging; MS, multiple sclerosis; NMOSD, neuromyelitis optica spectrum disorders; non-ON, non-optic neuritis; NPSLE, neuropsychiatric systemic lupus erythematosus; NS, neurosarcoidosis; ON, optic neuritis; OCT, optical coherence tomography; PMS, progressive multiple sclerosis; QI, quality index; RNFL, retinal nerve fiber layer; RRMS, relapsing-remitting multiple sclerosis; SD-OCT, spectral domain optical coherence tomography; SLE, systemic lupus erythematosus; VEP, visual evoked potential.
