# Supplementary material for: The Role of Optical Coherence Tomography in Differential Diagnosis of Multiple Sclerosis and Autoimmune Connective Tissue Diseases with CNS Involvement
Source: J Clin Med. 2020 May 21;9(5):1565. doi: 10.3390/jcm9051565 (PMC7290953; doi:10.3390/jcm9051565)
Supplement: Supplementary file 1 [file jcm-09-01565-s001.pdf]

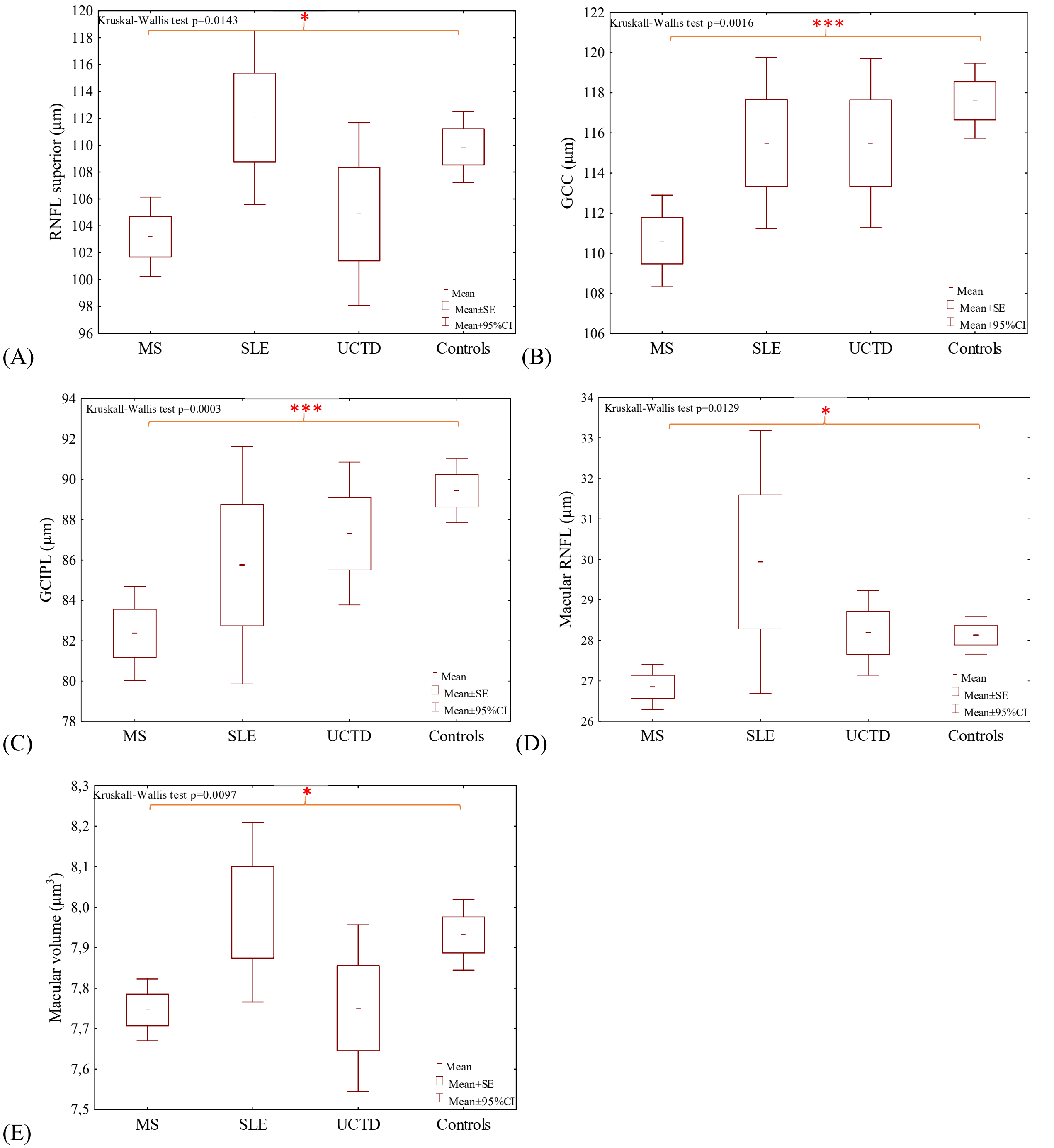

MS, multiple sclerosis; SLE, systemic lupus erythematosus; UCTD undifferentiated connective tissue disease; RNFL, retinal nerve fiber layer; GCC, ganglion cell complex; GCIPL, ganglion cell layer-inner plexiform layer

Figure S1. OCT parameters in the subgroups of CTD patients

There was a significant group effect with regard to superior optic disc RNFL (A), GCC (B), GCIPL (C), macular RNFL (D) thickness, and macular volume (E) (Kruskal-Wallis analysis p-values shown in the figure, respectively: 0.0143, 0.0016, 0.0003, 0.0129, 0.0097). Post-hoc analysis revealed that MS patients have significantly lower values of abovementioned parameters compared to healthy controls (respectively two-sided p-value with a Bonferroni adjustment: 0.0228, 0.0007, 0.0001, 0.0271, 0.0193). No significant group differences were observed between the patient groups (MS vs. SLE, MS vs. UCTD), nor between each CTD group (SLE, UCTD) and healthy controls. Statistically significant differences in the post-hoc analysis are indicated (*$p < 0.05$, ***$p < 0.001$).

	MS group Mean thickness, μm (SD)	Connective tissue diseases group Mean thickness, μm (SD)	Healthy control Mean thickness, μm (SD)	Kruskal-Wallis analysis p value
Optic disc RNFL average	90.00 (11.50)	92.14 (10.32)	94.63 (7.48)	0.0641
RNFL superior	103.55 (15.38)	106.07 (12.84)	109.83 (10.79)	0.0202
RNFL nasal	60.89 (11.74)	64.07 (11.76)	64.16 (10.35)	0.1369
RNFL inferior	104.62 (14.89)	105.95 (13.86)	109.47 (11.65)	0.2275
RNFL temporal	60.17 (12.45)	62.31 (11.83)	63.34 (9.35)	0.2234
Macular RFNL	110.86 (11.69)	114.16 (8.52)	117.61 (7.63)	0.0146
RNFL+GCL+IPL	82.55 (12.04)	86.02 (8.49)	89.44 (6.49)	0.0010
GCL+IPL	26.89 (2.88)	28.22 (4.03)	28.14 (1.88)	0.0002
Macular volume	7.76 (0.40)	7.80 (0.39)	7.93 (0.35)	0.0149

Mean results of OCT measurements (standard deviation)

Table S1. Mean values of OCT measurements

Mean values of OCT measurements: RNFL thickness, ganglion cell complex (GCC) thickness, ganglion cell layer-inner plexiform layer (GCIPL) thickness, and volume of the macula in non-optic neuritis eyes.
